# EnCOUNTer: a parsing tool to uncover the mature N-terminus of organelle-targeted proteins in complex samples

**DOI:** 10.1186/s12859-017-1595-y

**Published:** 2017-03-20

**Authors:** Willy Vincent Bienvenut, Jean-Pierre Scarpelli, Johan Dumestier, Thierry Meinnel, Carmela Giglione

**Affiliations:** Institute for Integrative Biology of the Cell (I2BC), CEA, CNRS, Univ. Paris-Sud, Université Paris Saclay, 91198 Gif-sur-Yvette Cedex, France

**Keywords:** N-terminal modifications, Protein maturation, Acetylation, Quantitation, Processing tool, Organelle proteins, Transit peptide, Cleavage site

## Abstract

**Background:**

Characterization of mature protein N-termini by large scale proteomics is challenging. This is especially true for proteins undergoing cleavage of transit peptides when they are targeted to specific organelles, such as mitochondria or chloroplast. Protein neo-N-termini can be located up to 100–150 amino acids downstream from the initiator methionine and are not easily predictable. Although some bioinformatics tools are available, they usually require extensive manual validation to identify the exact N-terminal position. The situation becomes even more complex when post-translational modifications take place at the neo-N-terminus. Although N-terminal acetylation occurs mostly in the cytosol, it is also observed in some organelles such as chloroplast. To date, no bioinformatics tool is available to define mature protein starting positions, the associated N-terminus acetylation status and/or yield for each proteoform. In this context, we have developed the EnCOUNTer tool (i) to score all characterized peptides using discriminating parameters to identify bona fide mature protein N-termini and (ii) to determine the N-terminus acetylation yield of the most reliable ones.

**Results:**

Based on large scale proteomics analyses using the SILProNAQ methodology, tandem mass spectrometry favoured the characterization of thousands of peptides. Data processing using the EnCOUNTer tool provided an efficient and rapid way to extract the most reliable mature protein N-termini. Selected peptides were subjected to N-terminus acetylation yield determination. In an *A. thaliana* cell lysate, 1232 distinct proteotypic N-termini were characterized of which 648 were located at the predicted protein N-terminus (position 1/2) and 584 were located further downstream (starting at position > 2). A large number of these N-termini were associated with various well-defined maturation processes occurring on organelle-targeted proteins (mitochondria, chloroplast and peroxisome), secreted proteins or membrane-targeted proteins. It was also possible to highlight some protein alternative starts, splicing variants or erroneous protein sequence predictions.

**Conclusions:**

The EnCOUNTer tool provides a unique way to extract accurately the most relevant mature proteins N-terminal peptides from large scale experimental datasets. Such data processing allows the identification of the exact N-terminus position and the associated acetylation yield.

**Electronic supplementary material:**

The online version of this article (doi:10.1186/s12859-017-1595-y) contains supplementary material, which is available to authorized users.

## Background

N-terminal acetylation (NTA) is one of the major protein modifications of the eukaryotic cytosol and occurs mainly co-translationally [1, 2]. In plants, most chloroplast proteins are encoded in the nucleus, translated in the cytosol and targeted to the chloroplast by a transit peptide that is cleaved upon arrival inside the organelle. Large scale analyses show that 20–40% of these proteins are N-acetylated in their mature chloroplastic form [3, 4]. The determination of the associated cleavage site of the transit peptide (TP) are still challenging. The cleavage positions of the mitochondrial or plastid TP (mTP or cTP) can be predicted using TargetP or ChloroP softwares [5, 6], but the predictions are not always reliable [7]. Although experimental data provide useful information, it still remains difficult to identify the true N-terminal peptides amid the multitude of internal peptides identified in a large scale experiment. In addition, the determination of NTA yield is a difficult task with the tools currently available. As an exemple, Mascot Distiller (MD) allows NTA quantitation using peptides N-terminally labeled with d_3_- (heavy, H) or d_0_- (light, L) acetyl [8]. Although this tool was used to define Lys ε-acetylation yield [9], it is originally dedicated to provide protein differential quantitation. The determination of the NTA yield for each proteoform (especially for the mitochondrial and the plastidic mature proteins) is not easily available and requires some additional processing [10].

Therefore, the development of a new tool designed to perform the extraction of the data computed by Mascot and Mascot-Distiller is required. The combination of the outputs must provide a list of the mature N-termini and the associated accurate NTA yields. Although some alternative tools could be able to perform H/L ratio quantitation such as MaxQuant, the EnCOUNTer script is not able, presently, to handle other input file format than the Mascot and Mascot-Distiller ones.

The EnCOUNTer tool (Extraction and Calculation Of Unbiased N-Termini) uses a stepwise approach. First, the characterized peptides are scored to discriminate between protein N-termini at position 1–2 and downstream N-termini (DNT) of the protein sequence. This determination is based on a curated experimental dataset. Second, EnCOUNTer recalculates the average NTA yield taking into account the first residue of the characterised mature proteins. Finally, it provides an exhaustive list of the processed N-termini with the recalculated unbiased NTA yield. The EnCOUNTer tools was trained using a manually validated dataset (Additional file 1: Table S1). As a proof of concept, the optimized parameters were used against a complex *Arabidopsis thaliana* experimental dataset obtained after an enrichment of the mature protein N-termini using the SILProNAQ approach [2]. Such experimental data set provides 584 DNT peptides (related to 383 distinct proteins) of which 338 were quantified for NTA yield. Some of these N-termini (112 hits), were experimentally validated and their positions well correlated with known cleavage sites of signal peptides, mTPs or cTPs (based on UniProtKB/Swiss-Prot annotations). Some others (224 hits) were in accordance with transit peptide cleavage site predictions within a range of ± 2 residues. In addition, 648 protein N-termini were characterized at positions 1 or 2 (on the initiator methionine or after its excision) of which 303 were also quantified for protein NTA yield (Additional file 2: Table S2).

## Methods

### Sample preparation and raw data aquisition

Proteins extracted from *A. thaliana* Col. 0 seedling were used to perform N-terminus enrichment using SCX chromatography. Rapidly, 1 mg of protein was denatured and reduced followed by cysteine alkylation with iodoacetamide. After cold acetone precipitation, proteins were resuspended in 50 mM NH_4_HCO_3_ and digested by 1/100 (w/w) of TPCK treated porcine trypsin (Sigma-Aldrich) for 1.5 h at 37 °C, twice. Peptides were desalted with Sep-Pak columns and the retained material was eluted with 80% acetonitrile (ACN), 0.1% TFA and then evaporated to dryness. The collected material was resuspended in Strong Cation eXchange (SCX) LC buffer (5 mM KH_2_PO_4_, 30% ACN and 0.05% formic acid) and injected into an Alliance HPLC system using a fluorimeter detector (Waters) equipped with polysulfoethyl A column (200 × 2.1 mm, 5 μm 200 Å; PolyLC, Colombia, MD). Peptides were eluted with a KCl gradient (SCX-LC buffer B: 350 mM KCl in SCX-LC buffer A; 0–5 min, 0% B; 15–40 min, 5–26% B; 40–45 min, 26–35% B). Fractions were collected every 2 min for 40 min and the solvent was evaporated to dryness before storage at −20 °C. Fractions eluted from SCX columns with retention times of 3 to 22 min were analyzed as previously described ^1^ with an Easy Nano-LC II (Thermo Scientific) coupled to a LTQ-Orbitrap™ Velos (Thermo Scientific). Finally, data processing usually combines a few acquisition files, i.e. 10 files related to each individual SCX fraction (1 h analysis) and 6 files related to combined fractions (for more details, see [2]). Furthermore, acquisition files obtained from SCX fraction 5 and 6 were used as training dataset and testing dataset, respectively.

### Mascot Distiller/Mascot data processing and *.xml exports

**Fig. 1:**
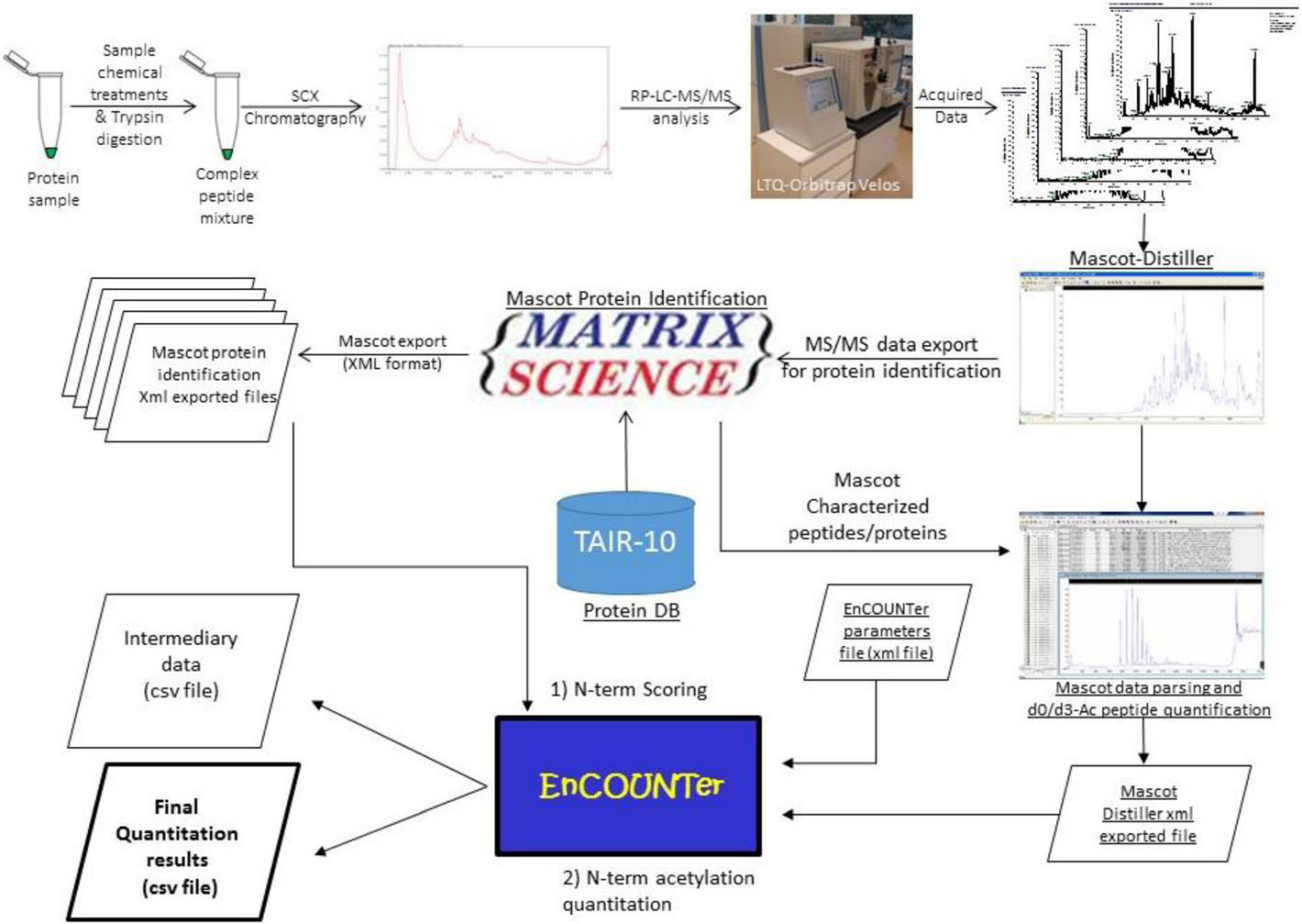
Overview of the EnCOUNTer processing scheme: from sample to mature N-terminus position and NTA yield

Regardless of the number of Orbitrap-MS acquired files, Mascot Distiller (Ver. 2.5.1, Matrix Science, London, UK) combines all acquired files together for a unique processing event (Fig. 1). The EnCOUNTer script was first used to extract data from the raw files followed by protein identification using the Mascot protein identification tool (Ver 2.4, Matrix Science, London, UK). Precursor detection was obtained after peak re-gridding of 100 points per Da for a peak half width of 0.02 Da. Precursor ion charge was determined directly from the Orbitrap survey scan and limited at 1 to 5. The “time domain aggregated MS/MS spectra” was re-gridded at 20 points per Da for a peak half width of 0.2 Da and MS/MS spectra containing more than 10 peaks were retained. MS/MS spectra of similar precursor masses were not combined at any time and considered separately for peptide identification. Additional filtering parameters such as correlation threshold and minimal signal over noise ratio were defined at 0.7 and 1, respectively for the MS and MS/MS in the relevant mass range (400–20000 Da and 50–10000 Da, respectively) with a maximum peak iteration of 500. Alternative high resolution mass spectrometers could be used but the MD parameters applied for raw data extraction should be optimized accordingly.

MD extracted data were submitted to Mascot 2.4 software for protein identification and post-translational modification characterization. The database used was “The Arabidopsis Information Resource” (TAIR ver. 10; www. arabidopsis.org [11]). The parent and fragment mass tolerance were 5 ppm and 0.4 Da, respectively. Additionally, carbamidomethylcysteine and d_3_-acetyl on Lys were defined as fix modifications and methionine oxidation as variable modification. Semi-trypsin was defined for the enzyme cleavage rule with up to 6 missed cleavages. Peptide N-terminus acetylation status, i.e. d3-Acetyl (chemically induced modification) or d0-Acetyl (endogenous modification) were investigated using the Mascot quantification option (associated to the MD parameters). These parameters (“Acetylation [MD]” quantification method) are available in Additional file 3. Then, MD uploaded the Mascot processing results and parsed them using relaxed parameters (minimum peptide identification score was set at 25, 0.2 for the P-value, 0.1 for the peak correlation coefficient, the area fraction coefficient and the precursor standard error). Irrelevant and false positive peptide hits generated at this step were filtered out at the final stage of the EnCOUNTer process.

EnCOUNTer also required protein identification data generated by Mascot. These data were automatically exported in xml files with the same MD parameters for the P-value and the Mascot score threshold. Additionally, the “MudPIT Scoring” and “Bold Red peptides” option were selected. These exported files contain all “Protein Hit Information” except pI and Taxonomy ID and all “Peptide Match Information” except the frame number and the unassigned queries.

### EnCOUNTer processing

Basically, the EnCOUNTer tool requires the MD exported file, the associated Mascot results and a parameter file. Although the tool could be used with the default parameters provided (Additional file 4), an optimization of the scoring parameters using a relevant training dataset has been performed. During the scoring parameter optimization, the EnCOUNTer tool required an additional files containing the list of “curated N-termini” (True / False N-termini; Additional file 1: Table S1). At the end of the optimization, a file containing all optimized values was generated (*.json). This file could be applied on other experimental datasets (from a similar origin) without the optimization of the scoring scheme.

#### Parsing function

The EnCOUNTer tool parses the pre-processed data exported from MD and Mascot identification tool. Mascot matched queries (only Mascot first-ranked peptide sequences) and associated protein AC were extracted from the MD xml file. Each of these entries were enriched with information, e.g. peptide sequence, starting position, MD processing results such as H/L ratio and signal quality coefficients. Then, the collected results were complemented with data extracted from the Mascot exported files such as the peptide identification score, identification E value… Of note, some peptides were not proteotypic [12] and shared with few distinct proteins or, alternatively, to different translational isoforms of the same protein (especially for TAIR database). The redundancy is noted and these data could be easily removed at will. Also, the shared peptides were distinctively labelled in the final result list.

#### N-terminus scoring function

The EnCOUNTer tool should discriminate internal peptides from the mature protein N-termini. Biological details associated to nuclear encoded mitochondrial/plastidic proteins TP such as sequence composition and average length [13–16] (also observed from experimental dataset [3, 17, 18]), highlighted some features useful to define relevant scoring coefficients (Additional file 5: Figure S1 and Additional file 6: Figure S2). To this end, we defined a scoring function based on six distinct coefficients related to i) peptide “starting position”, ii) residues around the “starting position”, iii) characterized N-terminal modifications, iv) alternative start positions at the vicinity of the “starting position”, v) matched peptide redundancies and finally iv) the “Localization” score. Some of these features could be optimized from the training dataset (such as “starting position” or the “residues around the starting position”) whereas some other should be defined by the users to valorize/penalize experimental observations (such as “data redundancy” or “multiple transit peptide cleavage sites”.

#### Peptide “starting position” score (Bound Score)

Based on the experimental training dataset, EnCOUNTer determines the optimal range (OptiMin and OptiMax) where “true” N-termini are the most frequently distributed. The Matthews Correlation Coefficient (MCC) was determined for all possible combinations of positions between the two endpoints of the N-terminal distribution range of the “True” hits for the DNT candidates (defined as ExpMin and ExpMax). The optimum range defined with the higher MCC provides the optimum endpoints (OptiMin and OptiMax). This positional range is associated with a scoring weight of 2 to favor the characterization of these N-termini. This calculation was associated to a “K fold cross validation” (using 10 randomized fractions) to determine the robustness of the prediction and the results of the investigation were exported in the *.bound file (specifically for the “bound” K fold test) and *.json (all optimized values).

Nevertheless, some relevant candidates (Experimental “True” N-termini) were still present outside of these optimal values, i.e. in between ExpMin/OptiMin and OptiMax/ExpMax. Since the experimental dataset may be slightly different compared to the training dataset (considering the ExpMin and ExpMax values extracted from the training dataset), the ExpMin and ExpMax values were pondered by the standard deviation observed during the “K fold cross correlation” as an estimation of the dataset variability (defined as Min and Max respectively). Both ranges, i.e. Min/OptiMin and OptiMax/Max, were associated with a scoring weight of 1 (neutral effect on the result) that prevented their elimination at this stage. All others positions are associated with a scoring weight below 1 (e.g. 0.1) to penalize such less biologically relevant positions. Starting positions 1–2 were subjected to a special scoring detailed below.

### Residues around the starting position (“Spec” Score)

Based on the training dataset, EnCOUNTer is able to provide a scoring matrix associated to the amino acid presents around the experimentally characterized starting position. For each position located between P_n_ to P_-n_, a binary classification was performed for each of the 21 possible amino acids. Such investigation provides a distribution of True Positive (True N-termini candidate has the defined residue at P_i_), True Negative (False N-termini candidate has not the defined residue at P_i_), False Positive (True N-termini candidate has not the defined residue at P_i_) and False Negative (False N-termini candidate has the defined residue at P_i_). The MCC was calculated for each of the 21 residues for each of the defined position between P_n_ to P_-n_. The result of the MCC provided an overview of the “abundance” for each residue at a specific position (P_i_) based on the training dataset. In accordance with our scoring scheme (1 for the neutral value), the MCC results were translated by 1 unit (tMCC). This tMCC matrix (defined for each of the 20 amino acids on a “±n” residues around the starting position of the peptides present in the training dataset) was used to determine the “Spec” score for each experimental candidate (with a starting position higher than 2) as a product of the tMCCs values for each position between P_-n_ to P_n_ (Eq. 1). The size of the screening window (±n) was also optimized automatically to determine the optimum MCC value.

1 $$ S p e\; Score={\displaystyle \prod_{i=- n}^n tMCC\left( Xxx; Pi\right)} $$

Determination of the “Spec” score base on the tMCC determined for each possible residue (Xxx) at the define position P_i_ (in the P_-n_ - P_n_ range).

A “K fold cross validation” (subdivided in 10 subsets) was applied after the optimization step to determine the robustness of the prediction. The “K fold cross validation” result was exported in the *.spec (specifically for the “Spec” K fold test) and *.json (all optimized values).

#### N-terminal modifications (Acetyl Score)

Due to the MD processing applied during the peptide identification step, peptide’s N-terminal modifications are restricted to d0/d3-NTA. Three different situations could occur (d0-NTA, d3-NTA and d0/d3-NTA). It could be interesting to segregate differentially such peptides especially for GAP test [19] where the main goal is to identify the N-terminal acetylated (NTAed) proteins and to rate them differently with values higher than 1 to valorize the modification or below 1 to penalize it. Characterization of the pair d0/d3-Ac reinforces the legacy of such N-termini the MS/MS spectra related to d0-Ac and d3-NTA could be considered as two independent events) and a score higher than 1 could be applied.

#### Alternative start positions (Prox score)

Despite proteins processing sites are usually considered to be unique, experimentally-based results tend to display a different reality involving multiple vicinal cleavage positions [7, 8, 17]. This provides clear and highly valuable distinctive criteria compared to internal protein fragments. The number of potential cleavage sites was determined in a defined window around the investigated position. Both the window size and the coefficient-weight could be defined in the parameters file (Additional file 4), and the “prox score” for this coefficient was obtained using Eq. 2. Initial parameters should be defined in the configuration file and optimized using the reference dataset but usually these multiple and vicinal cleavages [3, 17] are observed in a windows of ± 5–10 residues (defined at will in the parameter file).

2 $$ \mathrm{Prox}.\mathrm{Score}.={\mathrm{R}}^{\mathrm{m}} $$

With R = user defined weight and m = number of alternative cleavages sites experimentally characterized in the defined window (±5 residues range defined in the “Default parameters”);

#### Peptide redundancy (Rep Score)

Multiple characterization of the same peptide strengthens the probability to match a real event. Since each analysis provides thousands of acquired independent MS/MS spectra, the identification of the same peptide from different MS/MS acquisition could be considered as independent event and strongly increase the probability to match a “real event” or “true peptide”. To take advantage of this redundancy, the number of occurrences of the same peptide (not considering variable charge states or possible associated modifications such as Met oxidation) was used in the “Rep Score”. Nevertheless, the number of duplicates matches could reach few hundreds to few thousands of hits for the same peptide especially if multiple LC-MS acquisitions are processed together. To maintain the weight of this coefficient within the range compatible with the others scoring coefficients, the number of occurrences for identical peptides was logarithm pondered in Eq. 3.

3 $$ \mathrm{Rep}\ \mathrm{Score} = {\mathrm{K}}^{\log \left(\mathrm{q}\right)} $$

Where K is the score associated to such event (K = 2 is defined in the “Default parameters”) and q = number of experimental occurrences of the investigated starting position;

#### Localization score (Loc Score)

It is experimentally infrequent [2, 20, 21] to characterize mature protein N-termini both at the N-terminal side of the predicted protein (Pos 1–2) and further downstream in the same sample. Thus, it could be interesting to take advantage of such information to penalize/favor DNT peptides. The weight applied to DNT hits should be defined at will in the configuration file.

#### Protein N-terminal scoring at position 1–2

Since a negative dataset could not be defined for the N-termini at position 1 and 2, automated optimization of the score is not possible. The “Spec” score for these peptides is set at the optimized “Spec-score-threshold (automatically defined during parameters optimization) to favor the final NTA quantification of these peptides. To note, the other scoring coefficients (i.e. N-terminal modification characterized and peptide redundancy) were applied for these positions. Then, the final EnCOUNTer score for these peptides (Position 1–2) could not be compared with the DNT associated scores.

#### Scoring parameters optimization and calculation

Since few parameters such as sample preparation or species influencing the type and number of downstream N-termini (True or False hits), a test sample dataset should be used to optimize the parameters. Alternatively, default parameters are provided for the *A. thaliana* samples.

The EnCOUNTer tool is able to optimize few scoring coefficients (i.e. the optimum downstream N-termini range and the “vicinal cleavage site” scoring matrix) using a reference files. Some other parameters are not optimized automatically and could be subject to modification in the parameter file. Each of them (Prox, Rep and Loc scores) should be defined before the EnCOUNTer optimization to determine automatically the EnCOUNTer threshold. This threshold is optimized using an MCC approach at the end of the optimization step. Since the EnCOUNTer score is the product of the six previously defined coefficients (Eq. 4), each of them could be neutralized using the unit value (“1”) in the parameter files except for the “Spec” scoring coefficient which is the backbone of this approach. Scoring calculation was applied for each Mascot characterized peptide.

4 $$ \mathrm{EnCOUNTer}\ \mathrm{Score} = \mathrm{Bound} \times \mathrm{Spec} \times \mathrm{Acetyl} \times \mathrm{Prox} \times \mathrm{Rep} \times \mathrm{L}\mathrm{o}\mathrm{c} $$

This optimization finishes with a “K fold cross validation” to provide some insights about the prediction robustness and the results are saved in the *.score (specifically for the final EnCOUNTer score K fold test), *.json (all optimized values) and *.param (all parameters resumed) file.

#### NTA quantification function

Mascot Distiller is an interesting tool to determine d0/d3-Acetylation yield for each characterized peptide. Although, final quantitative values are provided per protein and not per protein starting position, the quantitative data remain available in the MD xml exported file. The EnCOUNTer script re-organizes them to provide N-terminal acetylation yield for each distinct proteoform. Since MD processing was performed using relaxed parameters (see Mascot Distiller data processing section), EnCOUNTer filters those data to retain only the most relevant ones for the NTA quantitation based on MS signal quality coefficients defined by MD. EnCOUNTer tool uses the “Correlation Coefficient” (related to the fitting between the theoretical and experimental isotopic distribution; higher than 0.8), the “Fraction Coefficient” (defining the fraction of the signal of the peak of interest over all signal; usually higher than 0.5), the SigQual coefficient is associated to the H/L standard deviation (defined by the least squares fit to the heavy vs. light component intensities from the scans in the XIC peak; lower than 0.05 Da), the E-value (lower than 0.05), the Mascot score associated to the matched query (higher than 30; highly dependent of the database used) and finally the EnCOUNTer score threshold (automatically defined as previously described). These coefficients could not be defined by default and are strongly related to the raw MS signal quality. They should be adapted accordingly to the instrument (MS and LC) used for sample separation and analysis. The characterised peptides passing those criteria were used to determine the final H/L ratio for each distinct protein positions based on a logarithmic means. Jointly, the logarithmic deviation (σ) of the NTA yield was determined to provide the minimum/maximum NTA range when more than one ratios were determined. Finally, the average NTA yield was obtained from the average H/L ratio using the Eq. 5 and the confidence interval (Min and Max NTA percentages) was defined by the Eqs. 6 and 7 respectively using the logarithmic divergence coefficient.

5 $$ \%\ \mathbf{N}\mathbf{T}\mathbf{A} = 1\ /\ \left(1 + <\mathrm{H}/\mathrm{L}\ \mathrm{ratio}>\right) $$

6 $$ \%\ \mathbf{N}\mathbf{T}{\mathbf{A}}_{\mathbf{Min}} = 1\ /\ \left(1 + <\mathrm{H}/\mathrm{L}\ \mathrm{ratio} > \times \upsigma \right) $$

7 $$ \%\ \mathbf{N}\mathbf{T}{\mathbf{A}}_{\mathbf{Max}} = 1\ /\ \left(1 + <\mathrm{H}/\mathrm{L}\ \mathrm{ratio}>/\upsigma \right) $$

#### EnCOUNTer data export

The final results were exported in a *.csv file providing protein AC’s, the proteotypicity, the starting position, the N-terminal modifications characterised, the mature N-terminal sequence (first 10 residues after the starting position), the EnCOUNTer score, the < H/L > ratio (and deviation), the N-terminus acetylation yield (Average, Min and Max values). An additional file was also exported containing all collected and processed data (EnCOUNTer Intermediary file).

#### Training and testing dataset

An experimental dataset collected during a large scale *A. thaliana* N-terminome characterization was used for the optimizing and the testing steps. Out of the 16 acquired files, data associated to fraction 5 and 6 were used as training and testing datasets, respectively. First, the acquired data were processed for protein/peptide identification as described in “Distiller/Mascot data processing” section. For the peptides associated to a unique gene-ID but few different translation versions, the lowest TAIR extension number was retained in the final list. Non-proteotypic peptides (*i.e*., peptide matching several distinct gene-IDs) were removed from the final list. Each characterized peptide was manually checked to identify mature N-termini. Information from specialized databases such as AT_Chloro [22, 23], PPDB [24], SUBA [25], TopFind [26], MASCP-Gator [27] and various prediction tools such as TargetP / ChloroP / SignalP [5, 6]) or Mitofate [28] were used to assess N-termini relevancy and protein sub-cellular localization for each candidate (Additional file 1: Table S1). A total of 784 and 1006 checked peptides were dispatched in few different subcategories (Table 1 and Additional file 1: Table S1) in Fraction 5 and 6, respectively.

**Table 1:** Distribution of the manually checked peptides for the training and testing datasets (Fraction 5 and Fraction 6 respectively; based on Additional file 1: Table S1)

Starting position	Classification	Hits for Fraction 5	Hits for Fraction 6
Position 1 and 2	True Protein N-termini	189	261
	False Protein N-termini	0	1
Position > 2	True downstream N-termini	202	232
	Ambiguous downstream N-termini	63	61
	False downstream N-termini	329	451

#### EnCOUNTer Launch

The EnCOUNTer script should be launched in a prompt windows associated with the required files (fully described in the help support and the user manual). First, EnCOUNTer determined the optimized scoring parameters using the training dataset (MD and Mascot exported files) and the reference N-terminal list. A few files are exported at the end of the optimization including the optimized “scoring parameter” (*.json file) and the detailed results of the optimization and “K fold cross validation” (*.bound, *.param, *.score and *.spec). Second, the experimental datasets (MD and Mascot exported files) were scored using the previously optimized parameters to discriminate and quantify the mature N-termini and associated NTA yield. At the end of the process, the EnCOUNTer script provided two distinct files, i.e. the intermediary and final EnCOUNTer results. The intermediary file provided the detailed values used to determine the EnCOUNTer score and the individual NTA quantitation, whereas the final Encounter file provided the aggregated results per distinct proteoforms (EnCOUNTer score and the final NTA yied).

## Results and discussion

### Training and testing datasets

Two experimental samples were defined as training and testing dataset i.e. fraction 5 and fraction 6 respectively. The peptides characterized after the Mascot identification step are filtered using few different Mascot–associated values using the peptide E-value and the minimum Mascot score defined in the configuration files. These thresholds should be adapted to reach 1% of False Discovery Rate (FDR) at the peptide level. Applying these thresholds, false positive identifications for the expected N-terminal peptide (position 1 and 2) were infrequent (Table 1). As an example, no false candidate was characterized in Fraction 5 and only one probable false hit was listed in Fraction 6 (Additional file 1: Table S1). For these starting positions, the associated localizations were mainly the cytosol (49 hits), the membrane/vacuole (17 hits), the peroxisome (6 hits) or the mitochondria (without mTP, 5 hits). Only one plastidic protein (AT2G44640.1) was characterized with a mature N-termini at position 2. This infrequent but not unusual chloroplastic N-terminus [29] was confirmed experimentally and reported in PPDB [21]. The characterized N-termini at position 1–2 corresponded well to the expected cytoplasmic localizations.

Additionally, 595 peptides were characterized with downstream starting position (Start position > 2). These hits were sorted between True N-termini (mature protein N-termini; 203 hits), False N-termini (erroneous mature N-termini; 329 hits) and ambiguous N-termini (mainly poor MS/MS spectra quality or inconsistencies with previous biological and experimental facts; 63 hits). Only the True/False candidates were used during the EnCOUNTer training step. The main subcellular localization is the chloroplast with 73% of the candidates (149 hits) for the “True” dataset. Other locations such as cytosol, membrane or mitochondria were also found (21, 7 and 5%, respectively). At the contrary, the “False” dataset exhibits random location and similar distributions were also observed in Fraction 6 dataset (Table 1 and Additional file 1: Table S1). These two manually curated datasets (Fraction 5 and 6) were used during the EnCOUNTer training and testing steps, respectively.

### N-terminus scoring optimization

#### Residues around the starting position (“Spec” Score)

**Fig. 2:**
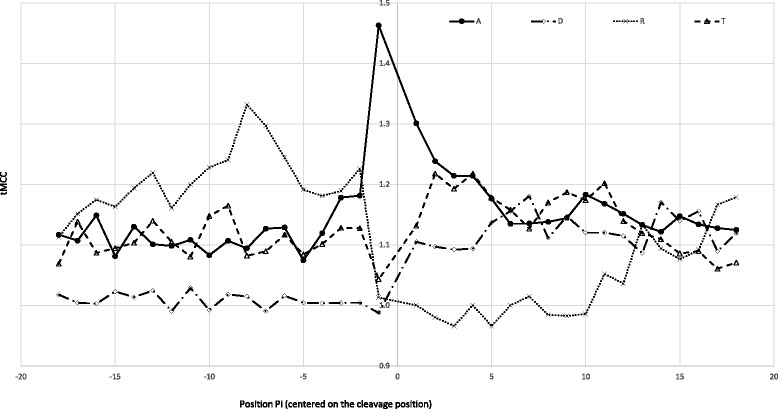
Example of the tMCC profiles related to few interesting residues around the transit peptide cleavage position

Residues close to the N-terminal position are, sometimes, associated to artifact modifications and/or (un)-expected endoproteolytic cleavages. As an example, hydroxylated residues (Ser, Thr or Tyr could be modified with a d3-NTA during sample preparation. Such modification located at P_1–3_ (see [30] for detailed positional nomenclature) could be wrongly associated to d3-NTA. The specificity of the endoproteinase used during sample preparation could also create a bias in the characterized peptides. Then, Arg residue at P’_1_ could be due to trypsin endoproteolytic cleavage (Additional file 7: Figure S3B) and may not be relevant as True maturation site. Along a streamline, the endoproteinases or the associated contaminants could generate numerous unexpected peptides. As an example, the presence of pseudotrypsin [31] or chymotyrypsin could generate some alternative N-termini with a Phe or Leu at P_−1_ [32]. Typically, it is well know that some positions ahead of the TP cleaving position could be specific such as Ala at P_−1_ and Val/Ile at P_−3_ for the plastidic proteins (Additional file 6: Figure S2A-B and [3, 21]) or the Arg at P_−2_ and P_−3_ for mitochondrial proteins (Additional file 6: Figure S2C-D and [16]). Interestingly, different residues appear to be predominant for other protein subclasses, for example Leu at position P_−9_ or Asp at P_1–3_ for proteins carrying a signal peptide (Fig. 2 and Additional file 6: Figure S2). The tMCC profiles (distribution of tMCC values at position P_-n_ to P_n_ for each distinct residue) clearly reflect the importance of these residues (Fig. 2 and Additional file 7: Figure S3) used in the “Spec” score.

The “Spec” coefficient is determined using a weight matrix of based on the distribution of specific residues around the cleavage site using the MCCs. MCC reflects the presence/lack of specific residues around the cleavage position. This determination can be performed for both the “True” and the “False” reference dataset (Additional file 7: Figure S3A and B) compared to a random distribution of transit peptide cleavage position (Additional file 7: Figure S3C). “Spec” score is the main basis of EnCOUNTer scoring scheme and could be used alone to determine the final EnCOUNTer score. The Spec-associated matrix was determined for both the “True” and the “False” subsets from Fraction 5 dataset. Based on the “True” hits, EnCOUNTer allows a discrimination at 94.0% accuracy and 97.6% specificity with 4.3% FDR whereas the optimization based on the “False” dataset reached only 88.5% accuracy and 91.6% specificity with 5.6% FDR (Table 2 and Additional file 8: Table S3). Only the optimization using the true hits is retained for the final scoring scheme.

**Table 2:** Results of the automated optimisation of the Bound and Spec parameters using the K fold cross validation result (*n* = 10) and the final scoring scheme using the same validation approach

Investigated parameters	Dataset or Scoring Scheme	ExpMin Position	ExpMax Position	EnCOUNTer or Spec threshold	True Positive	True Negative	False Positive	False Negative	Accuracy	Sensitivity	Specificity	False Discovery Rate	MCC
Spec (True dataset)	Training	-	-	> 62.9 ± 2.0	162 ± 3	288 ± 3	7 ± 2	21 ± 2	94.0 ± 0.3%	88.5 ± 1.0%	97.4 ± 0.5%	4.6 ± 0.9%	0.87 ± 0.01
	Validation	-	-	N.R.	16 ± 2	31 ± 3	2 ± 2	4 ± 2	88.5 ± 4.1%	78.7 ± 7.3%	94.9 ± 4.2%	9.3 ± 7.7%	0.67 ± 0.11
Bound (True dataset)	Training	17 ± 4	80 ± 6	-	164 ± 4	241 ± 6	56 ± 6	19 ± 3	84.3 ± 1.5%	89.8 ± 0.7%	81.2 ± 1.8%	25.3 ± 1.6%	0.71 ± 0.01
	Validation	N.R.	N.R.	-	18 ± 3	26 ± 4	7 ± 3	3 ± 1	82.9 ± 5.5%	87.7 ± 4.3%	80.0 ± 7.8%	26.6 ± 8.7%	0.67 ± 0.09
	All data together	14	78	63.2	183	266	63	20	84.4%	90.1%	80.9%	25.6%	0.69
Spec / Bound / Prox (True dataset)	Training	17 ± 4	80 ± 6	> 129.9 ± 0.6	167 ± 4	293 ± 5	3 ± 1	16 ± 2	96.1 ± 0.6%	91.2 ± 0.6%	99.1 ± 0.2%	1.6 ± 0.3%	0.92 ± 0.01
	Validation	N.R.	N.R.	N.R.	19 ± 4	33 ± 5	0 ± 1	2 ± 2	95.9 ± 2.9%	91.1 ± 5.3%	98.7 ± 2.3%	1.9 ± 3.2%	0.91 ± 0.06
Spec / Bound / Prox (False dataset)	Training	86 ± 9	300 ± 1	< 69.3 ± 6.1 (*)	272 ± 5	108 ± 4	74 ± 3	24 ± 4	79.5 ± 0.5%	92.0 ± 1.2%	59.1 ± 1.7%	21.4 ± 0.6%	0.59 ± 0.01
	Validation	N.R.	N.R.	N.R.	30 ± 3	12 ± 3	9 ± 3	3 ± 3	78.0 ± 4.1%	90.5 ± 6.7%	58.1 ± 10.1%	22.0 ± 5.4%	0.55 ± 0.08
Fraction 5 dataset	True dataset (Spec only)	14	78	65.1	179	321	8	24	94.0%	88.2%	97.6%	4.3%	0.872
	True dataset (Spec, Bound)	14	78	112.8	180	326	3	23	95.1%	88.7%	99.1%	1.6%	0.897
	True dataset (Spec, Bound, Prox)	14	78	130.1	185	326	3	18	96.1%	91.1%	99.1%	1.6%	0.917
	False dataset (Spec Only)	79	300	72.2	285	185	17	44	88.5%	86.6%	91.6%	5.6%	0.767
	False dataset (Spec, Bound, Prox)	79	300	66.7	304	118	84	25	79.5%	92.4%	58.4%	21.6%	0.556
	False dataset (Stringent params)	14	78	133.8	186	326	3	17	96.2%	91.6%	99.1%	1.6%	0.921
Fraction 6 dataset	True dataset (Spec, Bound, Prox)	14	78	130.1	179	442	8	51	91.3%	77.8%	98.2%	4.3%	0.806

(*) for the prediction based on the False dataset, the EnCOUNTer score must be below the determined Threshold for the True hits

Finally, a K fold cross validation (k = 10) was performed to determine the robustness of this approach. The accuracy reach 88.5 ± 4.1% and 94.9 ± 4.2% sensitivity with 9.3 ± 7.7% FDR (Table 2 and Additional file 8: Table S3). Although additional features should be used to prevent the loss of “True” hits, the results obtained using only the “Spec” score are extremely promising.

#### Peptide “starting position” score (Bound Score)

For most proteins, the mature N-term position is located on the first two residues of the protein sequence (position 1–2). Nevertheless, some proteins N-termini could be located further downstream (Position > 2). For example, the mTP cleavage position is expected between positions 20–70 whereas for the position for the cTP of *A. thaliana* nuclear encoded proteins is expected between positions 40–70 [16, 33]. In our training datasets (Additional file 1: Table S1), the validated downstream starting positions were distributed from position 3 to 106 (defined as ExpMin and ExpMax) for the validated candidates (“True” dataset) vs. 4 to 1104 for the irrelevant candidates (False dataset).

Interestingly, few distinct TP regions (Additional file 5: Figure S1) could be highlighted and are associated with proteins carrying a signal peptide (positions between 20 to 35 [34, 35]), mitochondrial TP (between positions 25 to 65 [16, 28]) and plastidic TP (between positions 30 to 95 [18]). Comparatively, the starting positions of the “False” candidates were evenly distributed. Then, it is interesting to favor/penalize selected regions depending of the protein training set. This allows the EnCOUNTer tool to define the optimum range where mature N-terminal positions are characterized from the training dataset. The optimum range for Fraction 5 dataset is in between positions 14–78 with 84.4% accuracy and 80.9% specificity with 25.6%. FDR. The associated “K fold cross correlation” (K = 10) highlights the robustness of this determination (Additional file 8: Table S3). This parameter cannot be used alone but always in combination with the Spec score (at least). When combining “Spec” and “Bound” score, 95.1% sensitivity and 99.1% accuracy with 1.6% FDR are reached on Fraction 5 training dataset. Such combination clearly improved the EnCOUNTer discrimination power compared to the “Spec” score alone.

#### Influence of the other scoring coefficients

By default, reliable predictions are reached using the Spec score and the Bound score together (95.1% accuracy at 1.6% FDR on the training dataset). Nevertheless, it could be possible to improve the prediction specificity or sensitivity using the additional coefficients Acetyl, Prox, Rep and/or Loc. Depending on the coefficient applied, it was possible to improve the sensitivity or the specificity of the EnCOUNTer tool (data not shown). As an example, the combination of Spec, Bound and Prox coefficients provides a final 96.1% accuracy and 99.1% sensitivity with 1.6% FDR. The associated K fold cross validation (k = 10) was performed and provided 95.9 ± 2.9% accuracy and 98.7 ± 2.3% sensitivity at 1.9 ± 3.2% FDR (Table 2 and Additional file 8: Table S3).

Although, the overall accuracy could be improved using different scoring combinations this was usually detrimental to the sensitivity. Depending on the goal (sensitivity, accuracy, specificity), scoring coefficient combinations could be adapted to reach better result than those provided in Table 2 i.e. better accuracy or better sensitivity… In hour hands, the combination of the scoring coefficients Spec, Bound and Prox provides a good starting compromise (Table 2) that could be optimized at will. These optimized parameters were applied to the Fraction 6 training dataset and provided the discrimination of the N-termini at 91.3% of sensitivity and 98.2% of specificity (4.3% of FDR).

### Protein N-terminal Acetylation quantitation

As previously mentioned, MD could provide protein NTA quantitation regardless of the multiple protein proteoforms. This is the example for the protein At1g16080.1 of which four distinct N-terminal positions could be characterized (positions 42, 44, 45 and 48; Additional file 1: Table S1). MD gave a single NTA yield of 35.5% (Min = 0.4%; Max = 98.98%) whereas EnCOUNTer provided four distinct NTA yield, i.e. 100.0% (Min: 99.8%; Max + 100.0%), 29.5%, 42.4% (Min: 42.0%; Max + 42.8%) and 2.1% respectively for each proteoforms. Another frequent MD processing error is the aggregation of H/L value associated to internal peptides. As an example, the MD quantification of At2g16600.1 protein combines the NTA yield associated to position 2 and 21 for a final NTA yield of 99.2% (Min = 26.5%; Max = 100.0%) whereas EnCOUNTer quantify only the N-terminus at position 2 with 99.8% NTA (Min = 98.5%; Max = 100.0%). Furthermore, the EnCOUNTer score of the peptide starting at position 21 is below the EnCOUNTer threshold and is not considered as a significant N-terminus. It is clear that EnCOUNTer discriminates the different N-termini and provides the most accurate NTA yield for each of them with an error range below 1% in average (Additional file 1: Table S1).

### Example of application

As an example of application, our whole experimental dataset (N-terminus enriched fraction from A*. thaliana* leave lysate [2]) was processed using the optimized EnCOUNTer parameters. The parameters used where based on the results obtained during the optimization phase (Table 2), i.e. the combination of the Spec, Bound and Prox coefficients. 3964 potential N-termini were listed of which 1554 have an EnCOUNTer score higher than the threshold (EnCOUNTer Threshold = 130.1). After the removal of the non-proteotypic peptides, 1257 probable mature N-termini were listed of which 649 were located at position 1–2 and 608 at positions downstream of the protein N-terminus (Position >2). The NTA yield was determined for 594 N-termini (excluded none proteotypic N-termini) of which 275 were located at position 1–2 and 319 were associated to DNT (Additional file 2: Table S2).

**Fig. 3:**
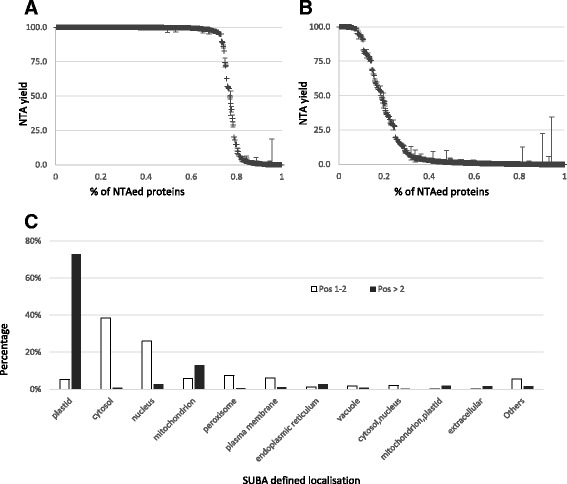
**a**-**b** Average NTA yield determined by EnCOUNTer for the N-termini at **a** Positions 1/2 of the characterized proteins and **b** downstream (Pos >2). **c** Protein subcellular localization distribution (SUBA based annotation [25]) for the N-termini position 1–2 and downstream

As previously observed [2], 73% of the characterised N-termini at position 1–2 were found fully acetylated (NTA > 95%), 18% not acetylated (NTA < 5%) and 9% were partially acetylated (Fig. 3a). These N-termini peptides were mainly located (Fig. 3c) in the cytosol (39%), the nucleus (26%) and also in the mitochondria (6%) and the plastid (5%). Protein located in these last two compartments are frequently associated to TP excision. Nevertheless, most of the characterised mitochondrial proteins are outer membrane proteins and the characterisation of a starting position 1 and 2 is biologically relevant. Similarly, 17 out of the 35 plastid proteins are coded by the plastid genome. These proteins, expressed directly in the plastid, do not undergo transit peptide excision and were expected in this subset. Considering the other 18 nucleus-encoded proteins which were annotated in SUBA (The SUBcellular localisation database for *A. thaliana* [25]) as being translocated to the chloroplast, some of them are known to be chloroplastic such as At2g44640.1 or At4g28440.1 [21] and the absence of cTP could be explained by some previously described alternative import mechanisms [29]. Other candidates are erroneously associated to plastidic localisation such as At5g24650.1 (TIM17) which is clearly a mitochondrial protein.

In addition to these expected N-termini, 608 downstream N-termini could be characterized with an EnCOUNTer score higher than the threshold of which 319 were quantified for NTA. The pattern of the DNT-NTA yield with 8% of the downstream N-termini fully acetylated (>95%), 25% partially NTAed and 67% not acetylated (<5%) was clearly different (Fig. 3b) from protein NTA profile (Fig. 3a). The subcellular distribution (Fig. 3c) was also strongly modified and the main localisation for the downstream N-termini was for 73% associated to plastidic proteins. Additionally, DNT also revealed mitochondrial N-termini (13%) resulting from mTP excision and alternative maturation of peroxisomal proteins (e.g. At2g33150.1 [36]), membrane proteins (e.g. At3g06035.1 or At5g19250.1 [37]) or vacuolar proteins (At5g60360.1 [38] or At2g23000). As previously observed for Pos 1–2, some of the SUBA subcellular localisation were erroneous, e.g. cytosolic localisation for At1G12900 or At4g26300 while they are localized in the chloroplastic stroma [39]. Some of the DNTs could also be a consequence of an alternative splicing or alternative start position (e.g. At1g66240 [40]), or errors on the gene starting position (At1g23820). Most of the 608 downstream N-termini highlighted by the EnCOUNTer tool were clearly due to protein maturation processes. This result confirms the added-value of EnCOUNTer to highlight mature proteins N-termini in complex peptide mixtures.

## Conclusions

Throughout few thousands experimentally characterised N-termini, the EnCOUNTer tool is able to parse the most relevant mature protein N-termini with 96.1% accuracy and 99.1% specificity on the training dataset (91.3% sensitivity and 98.2% specificity using Fraction 6 dataset). Furthermore, the EnCOUNTer tool is able to provide reliable NTA yield for each distinct proteoform at the expected protein N-terminus (Pos 1–2) but also downstream.

Applied to a large experimental dataset, the EnCOUNTer tool was able to characterize more than 1200 N-termini of which almost 600 were quantified for NTA yield. Those characterised DNT could be associated to different maturation processes including nuclear encoded proteins targeting to various organelles (e.g. mitochondria, chloroplast or peroxisome), cytosolic maturations involving transient targeting peptides (e.g. membrane or secreted proteins) or erroneously assigned protein starts. This tool provides a unique way to determine the experimental position of the protein mature N-terminus and NTA acetylation yield for few hundreds up to thousands of candidates. This tool is especially interesting to determine accurately and rapidly the influence of various stresses on protein N-terminal status and N-terminal modification yield.

## Additional files

Additional file 1: Table S1. List of the manually validated dataset (Fraction 5 and fraction 6) and curated subcellular localization. (XLSX 11079 kb)
Additional file 2: Table S2. Results obtained after EnCOUNTer processing applied to a large *A. thaliana* LC-MS/MS dataset (XLSX 381 kb)
Additional file 3: Mascot Distiller processing method for protein N-terminal acetylation quantitation. (PDF 24 kb)
Additional file 4: EnCOUNTer parameters file. (PDF 1 kb)
Additional file 5: Figure S1. Distribution of the transit peptide cleavage position for fraction 5 validated dataset (True and False) and few specific subset (Mitochondria, plastid…). (PDF 120 kb)
Additional file 6: Figure S2. Web logos and heatmaps of 231 of nuclear coded plastidic N-termini (A-B), 30 mitochondrial N-termini (C-D) and 35 N-termini associated to protein carrying an excised signal peptide (E-F). The sequences around the transit peptide cleavage are compared to the average distribution of *A. thaliana* using the stand alone ICELogo tool [41]. (PDF 474 kb)
Additional file 7: Figure S3. tMCC profiles of the Spec matrix for each possible residue for the A) True subset, B) False subset and C) from random A. thaliana proteins. (PDF 382 kb)
Additional file 8: Table S3. Result of the K fold cross validation (*n* = 10) for the optimization of bound, Spec and the global scoring using fraction 5 training dataset (True vs. False dataset). (XLSX 35 kb)

## Abbreviations

ACN: Acetonitrile
cTP: Plastid transit peptide
d0-Ac: endogeneous acetyl group
d3-Ac: deuterated acetyl group
DNT: Downstream N-termini
FDR: False Discovery Rate
H: Heavy (d3-Ac)
L: Light (d0-Ac)
LC: Liquid chromatography
MCC: Matthews Correlation Coefficient
MD: Mascot Distiller
MS: Mass spectrometry
mTP: Mitochondrial transit peptide
NTA: N-terminal acetylation
NTAed: N-terminal acetylated
SCX: Strong Cation eXchange
TFA: Trifluoroacetic acid
tMCC: translated (by 1 unit upper) Matthews Correlation Coefficient
TP: Transit peptide
