# Supplementary material for: EnCOUNTer: a parsing tool to uncover the mature N-terminus of organelle-targeted proteins in complex samples
Source: BMC Bioinformatics. 2017 Mar 20;18:182. doi: 10.1186/s12859-017-1595-y (PMC5359831; doi:10.1186/s12859-017-1595-y)
Supplement: Additional file 3: — Mascot Distiller processing method for protein N-terminal acetylation quantitation. (PDF 24 kb) [file 12859_2017_1595_MOESM3_ESM.pdf]

Quantitation Method: NAA Quant Meth

**Description**	Labeling of peptide N-term with light and heavy acetyl

Method

**Constrain Search**	yes
**Protein Ratio Type**	median
**Protein Score Type**	mudpit
**Report Detail**	yes
**Require bold red**	yes
**Minimum Peptides**	1

Protocol precursor

**Allow mass time match**	yes

Components

Component light

Modification Groups

Group Light acetyl

**Name**	Light acetyl
**Mode**	exclusive
**Required**	no
**Modification**	Acetyl (N-term)

Component heavy

Modification Groups

Group Heavy acetyl group

**Name**	Heavy acetyl group
**Mode**	exclusive
**Required**	no
**Modification**	Acetyl:2H(3) (N-term)

**Correction**	**Type :** averagine	
**Correction**	**Type :** impurity	**Element :** H 1
**Correction**	**Type :** impurity	**Element :** 2H 99

Ratios

Ratio H/L

**Numerator 1**	heavy	**Coefficient :** 1.0
**Denominator 1**	light	**Coefficient :** 1.0

Integration simpsons

**Integration Source**	survey
**Simple Ratio**	no
**Allow Elution Shift**	yes
**Elution Time Delta**	40 seconds
**Elution Profile Correlation Threshold**	0.15
**All Charge States**	yes
**All Charge States Threshold**	0.2
**Matched Rho**	0.5
**XIC Threshold**	0.1
**XIC Max Width**	50
**XIC Smoothing**	3

Quality

**Minimum precursor charge**	1
**Isolated Precursor**	Yes
**Minimum a(1)**	0.0
**Peptide Threshold Type**	minimum score
**Unique Pepseq**	no
**Isolated Precursor Threshold**	0.5

Outlier auto
