# Supplementary material for: EnCOUNTer: a parsing tool to uncover the mature N-terminus of organelle-targeted proteins in complex samples
Source: BMC Bioinformatics. 2017 Mar 20;18:182. doi: 10.1186/s12859-017-1595-y (PMC5359831; doi:10.1186/s12859-017-1595-y)
Supplement: Additional file 4: — EnCOUNTer parameters file. (PDF 1 kb) [file 12859_2017_1595_MOESM4_ESM.pdf]

```xml
<?xml version="1.0" encoding="UTF-8"?>
<config>
<Scores>
<Position P="2" R="1."/>
<Position P="Min" R="0.1"/>
<Position P="min" R="1."/>
<Position P="max" R="2."/>
<Position P="Max" R="1."/>
<Position P="inf" R="0.1"/>

<Loc F='2' R='1.'/>

<Acetyl V='Acetyl:2H(3) (N-term)' R='1.'/>
<Acetyl V='Acetyl (N-term)' R='1.'/>
<Acetyl V='-' R='1.'/>

<Prox range='5' R='2.'/>

<Rep R='1.'/>
</Scores>

<Filtre>
<E_value>0.05</E_value>
<Score>30</Score>
<Corr>0.8</Corr>
<SigQual>0.05</SigQual>
<Fraction>0.5</Fraction>
</Filtre>
</config>
```
