# Supplementary material for: EnCOUNTer: a parsing tool to uncover the mature N-terminus of organelle-targeted proteins in complex samples
Source: BMC Bioinformatics. 2017 Mar 20;18:182. doi: 10.1186/s12859-017-1595-y (PMC5359831; doi:10.1186/s12859-017-1595-y)
Supplement: Additional file 5: Figure S1. — Distribution of the transit peptide cleavage position for fraction 5 validated dataset (True and False) and few specific subset (Mitochondria, plastid…). (PDF 120 kb) [file 12859_2017_1595_MOESM5_ESM.pdf]

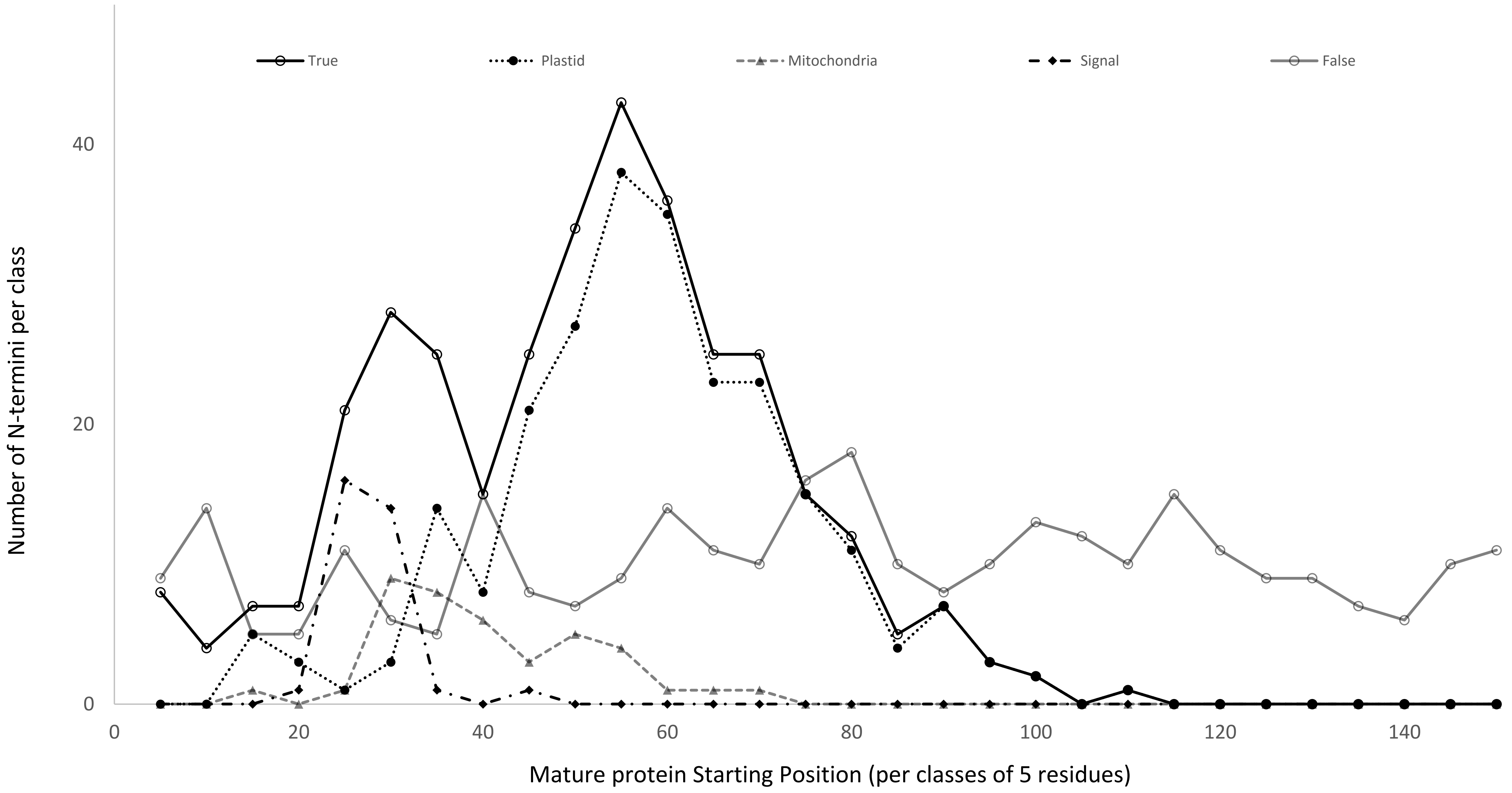

Additional file 5: Figure S1. Distribution of the characterized starting position including the True/False hits and various selected subsets such as plastidic, mitochondrial or signal peptide associated proteins.
