# Supplementary material for: EnCOUNTer: a parsing tool to uncover the mature N-terminus of organelle-targeted proteins in complex samples
Source: BMC Bioinformatics. 2017 Mar 20;18:182. doi: 10.1186/s12859-017-1595-y (PMC5359831; doi:10.1186/s12859-017-1595-y)
Supplement: Additional file 6: Figure S2. — Web logos and heatmaps of 231 of nuclear coded plastidic N-termini (A-B), 30 mitochondrial N-termini (C-D) and 35 N-termini associated to protein carrying an excised signal peptide (E-F). The sequences around the transit peptide cleavage are compared to the average distribution of A. thaliana using the stand alone ICELogo tool [41]. (PDF 474 kb) [file 12859_2017_1595_MOESM6_ESM.pdf]

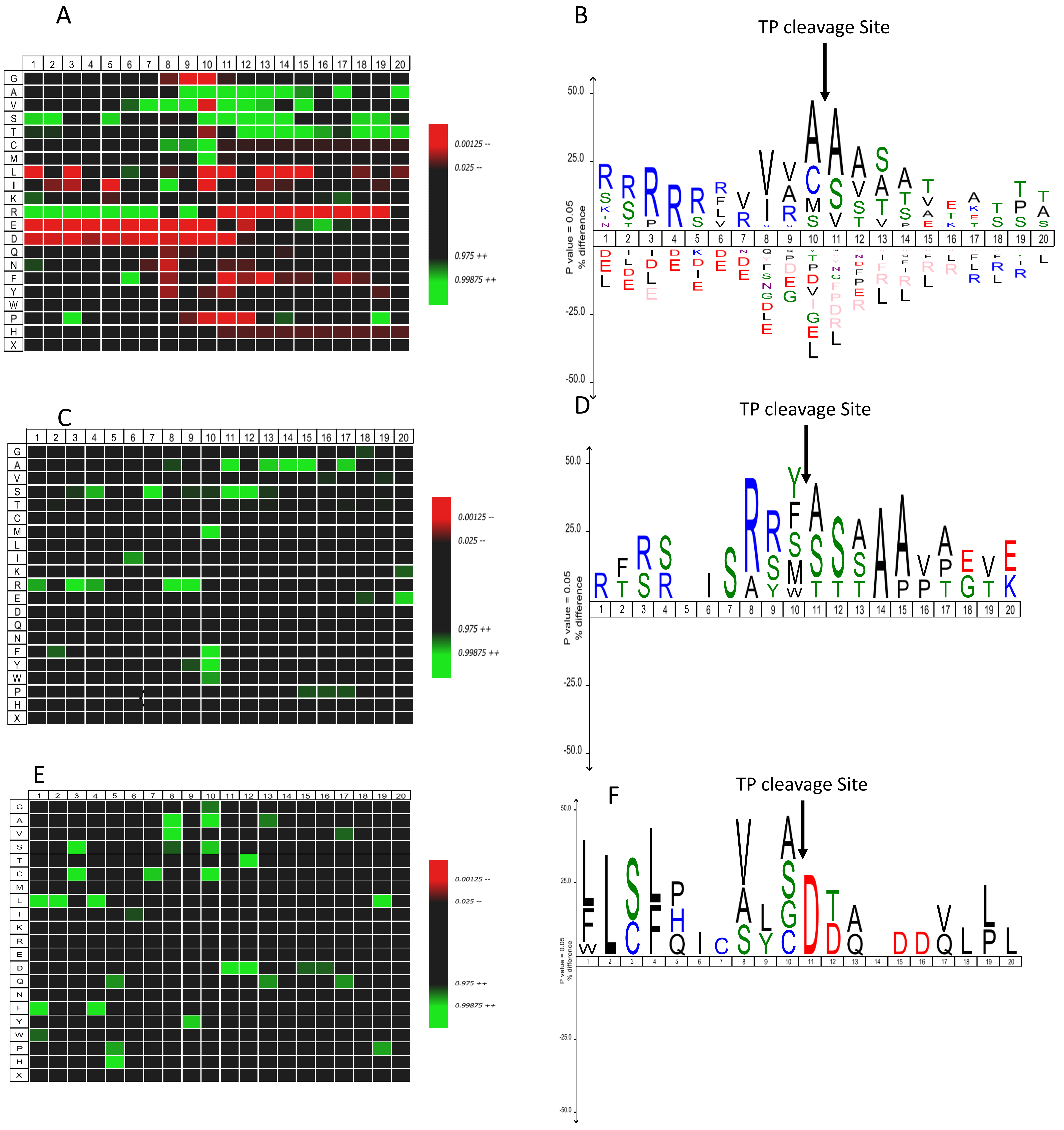

Additional file 6: Figure S2. Web logos and heatmaps of 231 of nuclear coded plastidic N-termini (A-B), 30 mitochondrial N-termini (C-D) and 35 N-termini associated to protein carrying an excised signal peptide (E-F). The sequences around the transit peptide cleavage are compared to the average distribution of *A. thaliana* using the stand alone ICELogo tool.
