# Supplementary material for: EnCOUNTer: a parsing tool to uncover the mature N-terminus of organelle-targeted proteins in complex samples
Source: BMC Bioinformatics. 2017 Mar 20;18:182. doi: 10.1186/s12859-017-1595-y (PMC5359831; doi:10.1186/s12859-017-1595-y)
Supplement: Additional file 7: Figure S3. — tMCC profiles of the Spec matrix for each possible residue for the A) True subset, B) False subset and C) from random A. thaliana proteins. (PDF 382 kb) [file 12859_2017_1595_MOESM7_ESM.pdf]

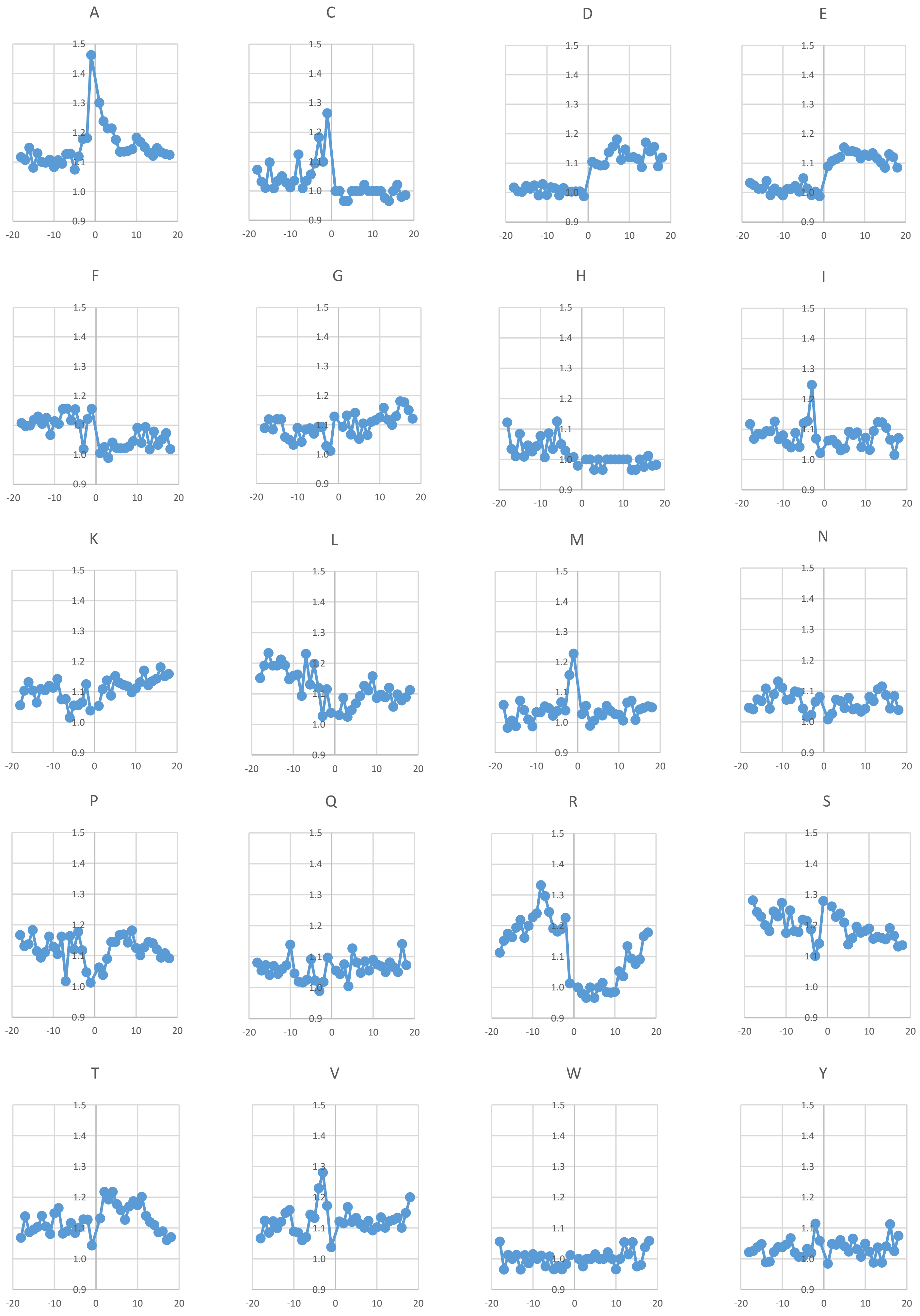

Additional file 7: Figure S3(A). tMCC profiles of the Spec matrix for each P_i positions for the curated True dataset.

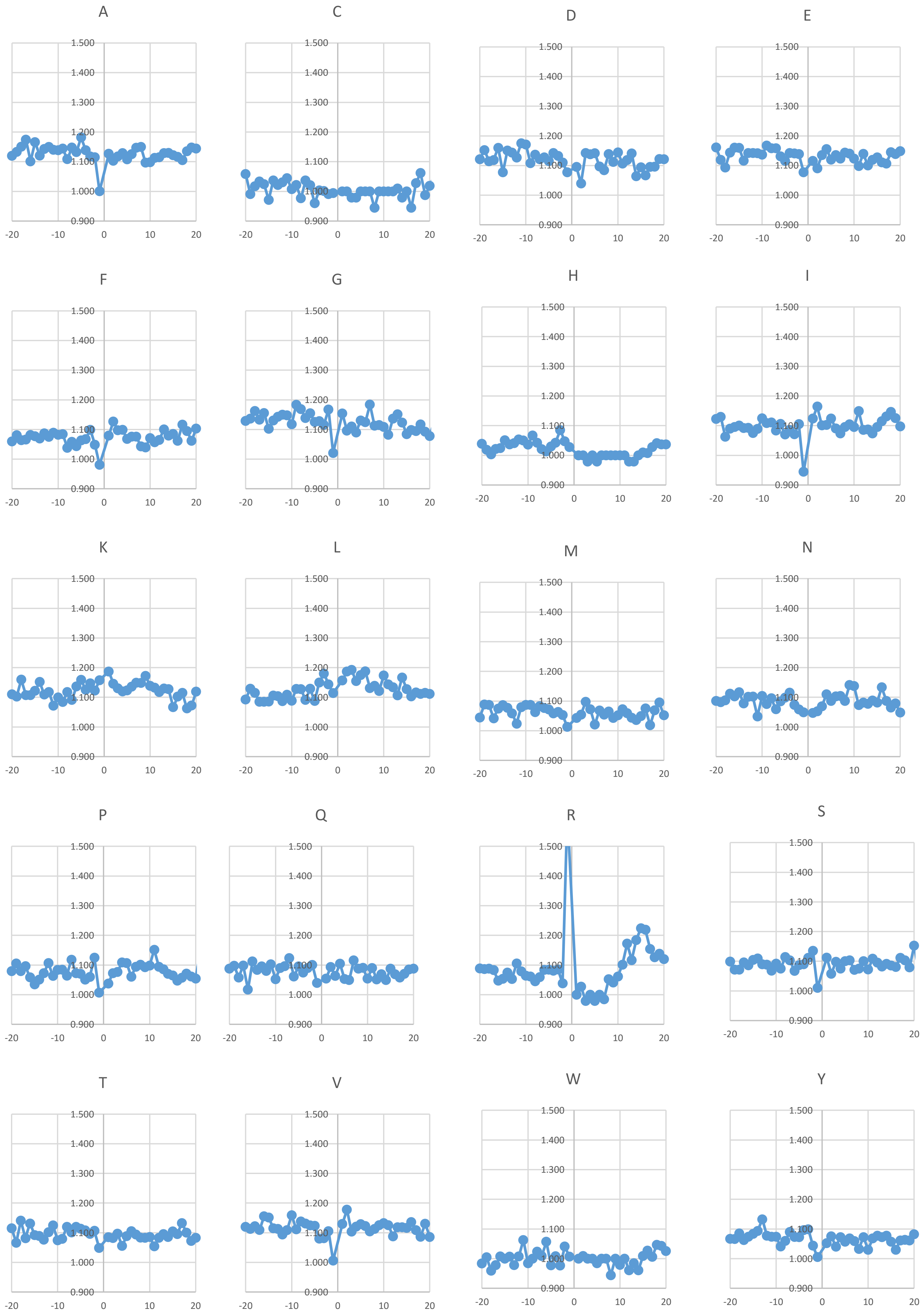

Additional file 7: Figure S3(B). tMCC profiles of the Spec matrix for each Pi positions for the curated False dataset.

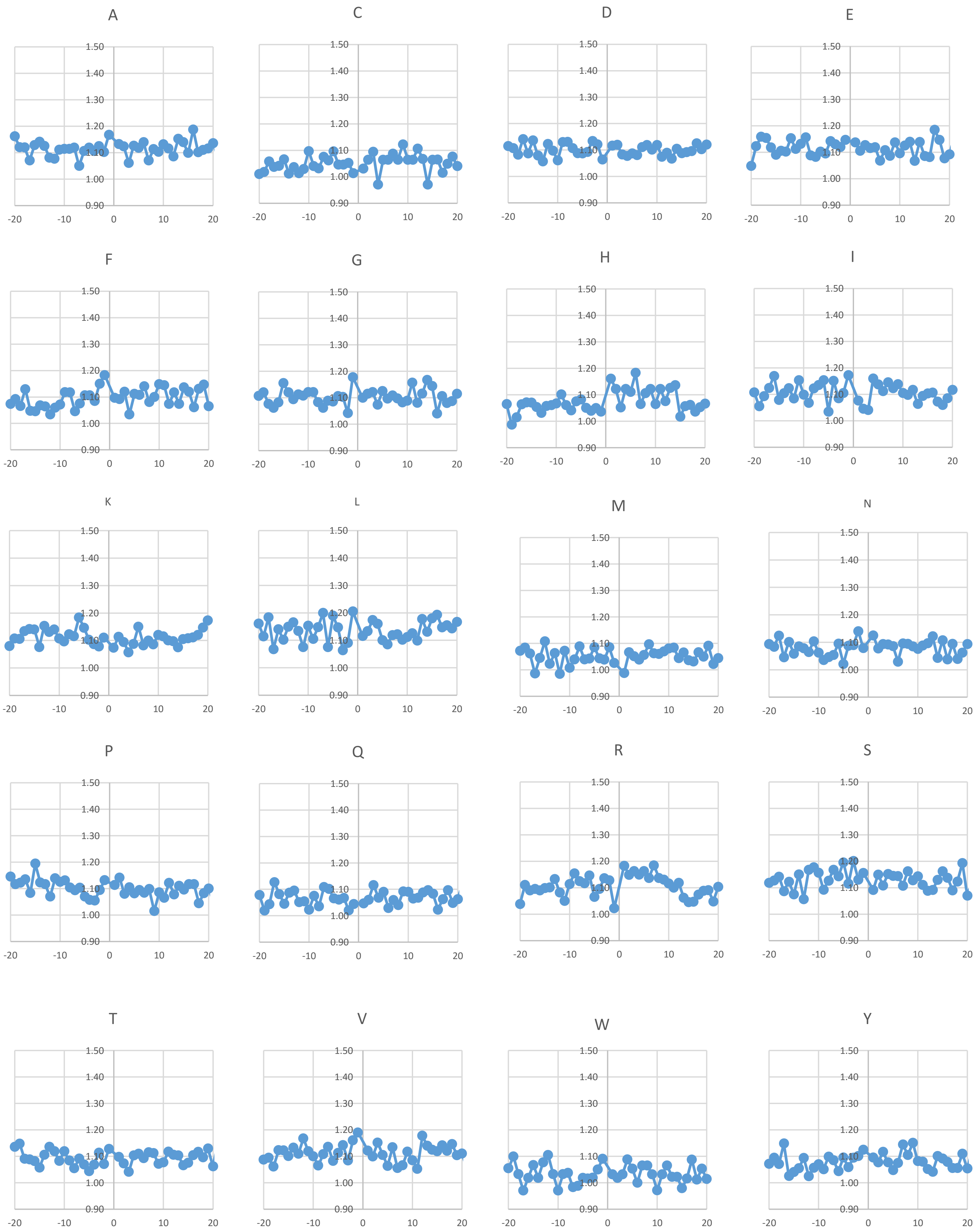

Additional file 7: Figure S3(C). tMCC profiles of the Spec matrix for each Pi positions for a random dataset.
